# Fictitious Rough Crack Model (FRCM): A Smeared Crack Modelling Approach to Account for Aggregate Interlock and Mixed Mode Fracture of Plain Concrete

**DOI:** 10.3390/ma13122774

**Published:** 2020-06-18

**Authors:** Jan Ungermann, Viviane Adam, Martin Classen

**Affiliations:** 1 Institute of Structural Concrete, RWTH Aachen University, 52074 Aachen, Germany; vadam@imb.rwth-aachen.de; 2 Department of Civil Engineering, KU Leuven, 2860 Sint-Katelijne-Waver, Belgium; martin.classen@kuleuven.be

**Keywords:** finite element analysis, 2D concrete material model, aggregate interlock, shear stress, concrete cracking, material model

## Abstract

The intention of this paper is to clarify the mechanisms of mixed mode fracture and shear stress transfer in plain concrete. To capture these scarcely explored phenomena, a new mechanical formulation is proposed called the fictitious rough crack model (FRCM). The FRCM considers mode I deformations to control crack formation and residual tensile stress transfer, while mode II deformations are assumed to induce shear stress transfer along the crack surfaces and compressive normal stresses attributed to aggregate interlock. The fundamental idea of the FRCM is to combine these tension-softening and shear-transfer laws and to superimpose the emerging shear and normal stresses of both mechanisms in the crack. The paper illustrates the analytical development of the FRCM and its numerical implementation. Three well-known experimental benchmark problems (concrete panel test series by Nooru-Mohamed and by Hassanzadeh as well as aggregate interlock test series by Paulay and Loeber) are numerically addressed to test plausibility of FRCM results. The numerical implementation of the FRCM is capable of simulating the transition from mode-I fracture to mixed-mode fracture in the structural response and is also able to predict the crack path with reasonable agreement.

## 1. Introduction

### 1.1. General

**Figure 1:**
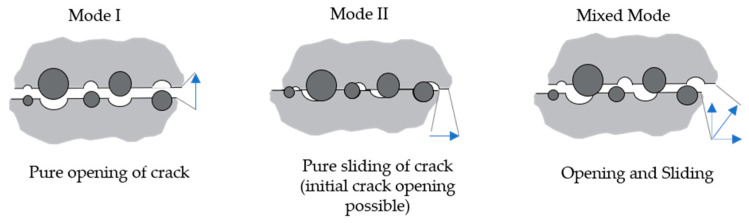
Illustration of the different crack modes according to Classen et al. [1].

The structural behaviour of plain concrete is significantly influenced by its quasi-brittle nature and the localisation and propagation of cracks. Concrete cracking is usually assumed to occur when the main tensile stress in the continuum exceeds the uniaxial or multiaxial tensile strength of the material. After initial concrete cracking, the kinematic behaviour of the crack faces can be defined by different modes, which are illustrated in Figure 1, namely mode I, often denoted as the opening or tensile mode, and mode II, which is denoted as sliding or shear mode. Furthermore, mixed mode is shown describing a kinematic process with co-occurring mode I and mode II displacements. Most fracture problems in concrete and reinforced concrete structures are of a mixed mode nature, involving perpendicular and parallel movements of the crack surfaces at the same time. Typical examples are shear and punching shear behaviour of reinforced concrete (RC) members. Despite its striking practical relevance, modelling shear and mixed-mode fracture is not yet well developed for cementitious materials. The following section gives a review of current modelling approaches to analyse the research significance.

### 1.2. Review of Existing Approaches to Model Tension and Shear in Cracked Concrete

#### 1.2.1. Mode I Behaviour

For many years, plenty of research has been devoted to the numerical representation of mode I fracture processes. With regard to the development of phenomenological models, the challenging task in continuum mechanics is to reproduce the process of crack localisation due to the growth and assembly of microcracks within the fracture process zone. In this context, models with smeared and discrete crack representation are distinguished.

Different smeared crack approaches were proposed of which the fictitious crack model by Hillerborg [2] had the most striking impact on the scientific community. Generally, the crack localisation is achieved by introducing strain softening material behaviour, representing the decreasing stress for an increasing strain along the coordinate normal to the propagating crack. However, this approach may lead to mesh-dependent results [3]. An additional treatment of the strain softening process using localisation limiters must be implemented to ensure objectivity of the model. An example of a regularisation technique often applied for damage-based models is the mesh-adjusted softening modulus that maintains the value of the energy dissipation constant (fracture energy) within a localisation crack band [4]. More advanced techniques apply the non-local averaging of state variables of a damaged or plasticity-based strain softening model.

It is important to note that the softening and crack localisation represent a process of evolving anisotropy, which must be captured by the orientation of failure planes. Different approaches are available to assess the stress and strain state in a material point in relation to the geometric framework. Some are described in the following. Besides fixed crack models where the crack is initiated perpendicularly to the maximum tensile stress after exceeding tensile strength, rotating crack models exist by accounting for secondary cracking by means of adjusting anisotropy [5]. Models based on the Microplane discretisation [6,7] pursue a different concept using an arbitrary number of potential cracking planes, which link the stress and strain space of a material point to the initial geometrical configuration of the represented material structure. Concrete damage is initiated when a local fracture criterion is violated, which may deviate from local mode I conditions. Besides regularised smeared crack approaches, the XFEM [8] method by Belytschko [9] is another possibility to obtain mesh independent results for the mode I fracture. Continuous re-meshing of the continuum enables these models to simulate a freely propagating discrete crack. In XFEM, the formulation of the crack tip element is of fundamental importance.

#### 1.2.2. Mode II Behaviour

Approaches to modelling aggregate interlock based on experimental investigations were presented for example by Paulay and Loeber [10], Taylor [11], and Bažant and Gambarova [12] (rough crack model). These were relating the normal and shear stresses to the crack opening and slip displacements. A fundamental mechanical analysis of this shear transfer mechanism (two phase model) was provided by Walraven [13,14]. However, the implementation of such approaches into numerical formulations is very scarce. In most smeared crack approaches, shear transfer in concrete after the initiation of concrete cracking is considered using rather simple empirical methods or is neglected entirely. In models considering this effect, a shear retention factor is usually applied, which was e.g., proposed by Cervenka [15]. In this case, the incapability of mechanically capturing aggregate interlock is addressed by means of reducing shear stiffness in the direction parallel to a fictitious crack. In Equation (1), $\beta_{s}$ = 0 represents the intact material while $\beta_{s}$ = 1.0 leads to a complete loss of shear transfer capability.

(1) $$G=\left(1-\beta_{s}\right)\cdot G_{el}.$$

Various numerical parametric studies aim at calibrating adequate shear retention factors for specific cases. Additionally, Scotta introduced approaches with variable shear retention factors [16]. However, this heuristic procedure is only to a certain extendable to accurately describe shear and mixed mode fracture of concrete under arbitrary loading conditions.

As mentioned before, the accurate formulation of crack tip elements is crucial in terms of modelling discrete cracks in XFEM. Most types of crack tip elements only feature simple constitutive laws for mode I fracture [17]. However, leading mode II and mixed mode are considered by decreasing stiffnesses depending on the crack width [18] or are even neglected. Therefore, these models are neither able to allow for crack propagation under mixed mode conditions nor to account for aggregate interlocking along the crack faces apart from the fracture process zone.

#### 1.2.3. Mixed Mode Behaviour

**Figure 2:**
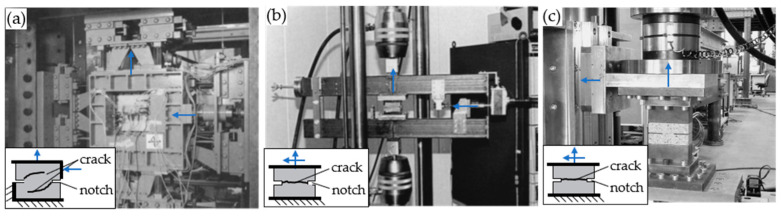
Mixed mode test setups by (**a**) Nooru-Mohamed [20] (1992), (**b**) Hassanzadeh [19] (1992), and (**c**) Jacobsen [21] (2012).

While crack propagation underlying mixed mode conditions can be observed in many practical problems (e.g., shear and punching). It is, however, challenging to experimentally isolate the pertaining effects for characterising adequate constitutive relations for a mixed mode fracture. Since the required test setups are material consuming and massive in order to provide sufficient stiffness for analysing mixed mode characteristics in concrete, only a few experimental studies are documented. Significant contributions to the understanding of mixed mode fracture in concrete were made by Hassanzadeh [19] (1992) and Nooru-Mohamed [20] (1992). Their test setups are illustrated in Figure 2a,b.

Mixed mode behaviour in smeared crack approaches (FE modelling) is usually represented by considering a material formulation combining the fictitious crack model (mode I) with a shear retention factor (mode II). The shortcomings resulting from the use of shear retention factors were already discussed. Additionally, interface elements can be used, which enhance the fictitious crack model through shear parameters [3,22,23,24] by assuming cohesive behaviour in mode I and mode II directions in cases where the crack path is already known. To this end, mode I and mode II fracture energies are required. However, the fact that some authors postulate mode II fracture energy to be negligible or non-existent [25,26] under mixed mode conditions, while others claim it to range between 10 [27] to 25 times [28] the mode I fracture energy, exemplifies the inconsistency of such approaches. Furthermore, the apriori definition of the elements’ position can lead to unrealistic results when choosing an inadequate initial crack location.

### 1.3. Benchmark Analysis for Selected Models

Even if there are various models [29,30,31] available to reproduce the numerical crack pattern of the mixed-mode tests of Nooru-Mohamed [20], the review of existing approaches to model tension and shear in cracked concrete clarified that aggregate interlock phenomena are rarely considered in current approaches. In the following, frequently used concrete models provided by different commercial software packages are applied for simulating Paulay’s [10] aggregate interlock experiments to evaluate the models’ ability to capture mode II behaviour. The following widely used models are analysed:

- Abaqus concrete damaged plasticity (CDP)
- ATENA constitutive model SBETA (CCSbetaMaterial)
- ATENA microplane material model (CCMicroplane4)

Selected properties of these material models are composed and evaluated in Table 1 based on the following theoretical principles and the results of the benchmark test. The smeared crack approach of CDP [8] includes a flow rule separating elastic and plastic stresses and strains. While mode I behaviour is considered by Hillerborg’s fictitious crack model, CDP does not account for any shear transfer across smeared cracks under mode II deformations. The plane stress state model SBETA [32] uses a variable shear retention factor based on the crack width for a simplified consideration of shear stresses due to aggregate interlock. SBETA offers both a definition of either a rotating or a fixed crack model. In contrast, the ATENA microplane model (M4) is based on strains and stresses on arbitrary planes [6,7,32]. In this case, the frictional response can be controlled by using fixed parameters for initial cohesion, which decrease depending on the volume expansion [7].

**Figure 3:**
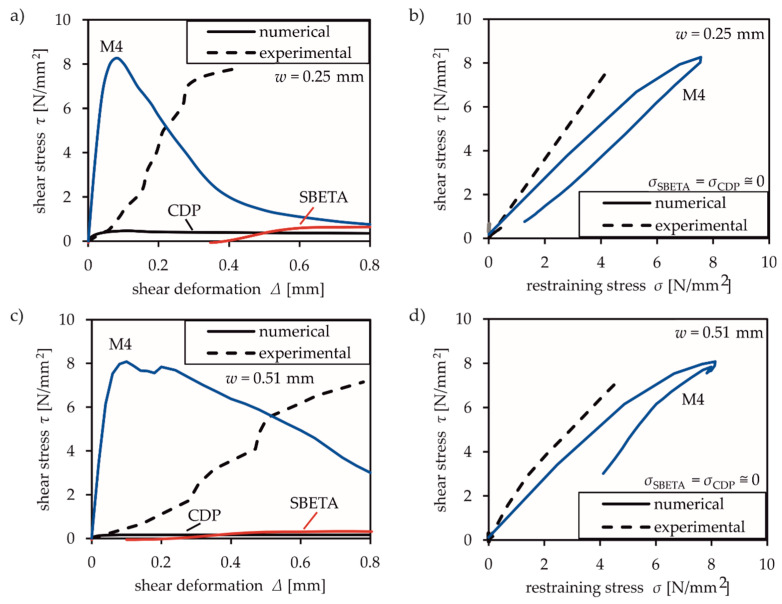
Comparison of experimental and numerical results based on tests by Paulay and Loeber [10] with constant crack width *w*: (**a**,**c**) shear stress-shear deformation curve and (**b**,**d**) shear stress-normal (restraining) stress relationship.

Figure 3 shows the comparison of the test results for a constant crack width of either 0.25 mm or 0.51 mm with the numerical results of the selected models. The numerical results were simulated as described in Section 3.3 and standard model parameters according to Table 2, Table 3 and Table 4. These evaluations are intended to provide a fundamental review of the ability to model mixed-mode tests. Several factors can be calibrated, especially when using the microplane model, but these do not contribute toward assessing the suitability of the models.

Clearly, CDP and the SBETA model significantly underestimate the transferable shear stresses across cracks (cf. Figure 3a,c). The resulting stresses according to the application of CDP are based on the rotating crack model. Despite considering transfer of increasing shear stresses at small crack widths (*w* = 0.25 mm) compared to larger ones (*w* = 0.51 mm), the overall accuracy of the simulation is very poor. The same applies to SBETA, which considers the transferable shear stresses by a shear retention factor of shear modulus. In Figure 3a,c, the numerical shear stresses of the SBETA model start at negative values and increase with growing shear deformation. The shear strength is limited by the maximum value of *f′*_t_ [32]. Both models (CDP and SBETA) seem to be inadequate in terms of capturing shear transfer across cracks. In addition, both models entirely disregard the occurrence of normal (restraint) stresses in Figure 3b,d.

The illustration of Paulay and Loebers’ experimental results with microplanes is significantly more accurate, especially regarding shear stress-restraining stress relationships in Figure 3b,d. The simulation of the shear stress-deformation curve distinctly deviates from test results. This model is based on material laws in normal direction and friction boundaries in shear direction on arbitrary planes with various orientations [6,7], but does not include an appropriate aggregate interlock law. Clearly, the interaction of normal and shear deformation and their influence on the resulting stresses cannot be appropriately modelled with normal stresses (compression and tensile laws) on arbitrary planes alone.

Conclusively, an accurate description of aggregate interlock effects is not possible with the well-known models described above. This is the motivation for aiming at improved modelling approaches that resulted in developing fictitious rough crack model (FRCM).

### 1.4. Research Significance and Scope of this Paper

In the past, large efforts were made to numerically implement mode I fracture processes while representing shear and mixed-mode behaviour attracted much less attention in the scientific community. Thus, the intention is to improve the understanding of crack mechanics and to clarify the open questions depending on the numerical consideration of the effects resulting from aggregate interlock. As shown in Section 1.3, especially the interaction of the crack opening and the shear deformation that is of crucial significance for the structural behaviour, which can neither be captured realistically by a simplified approach using a linear reduction of the shear modulus nor using micro planes.

**Figure 4:**
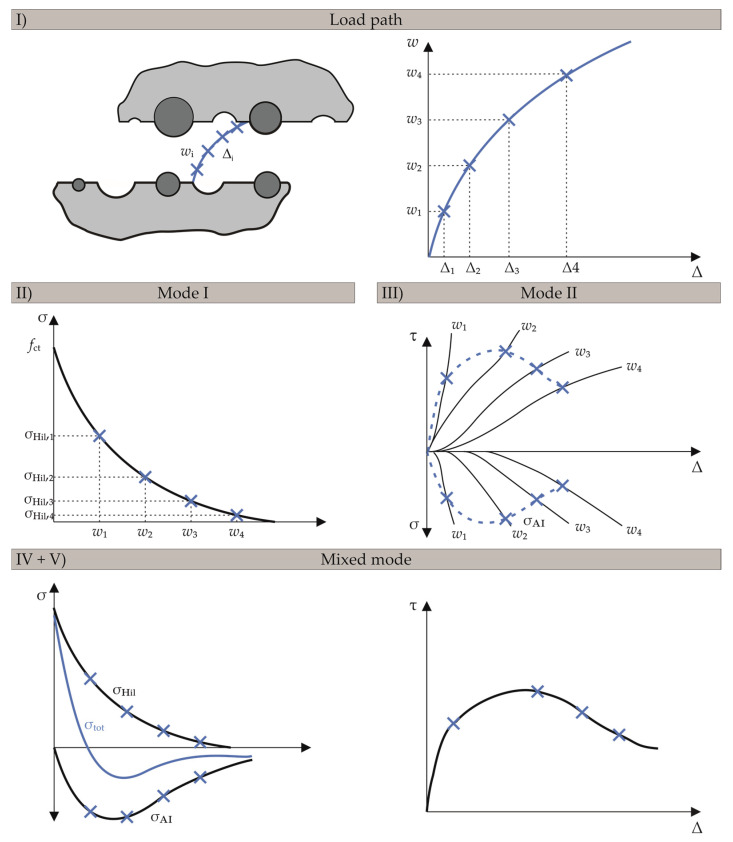
Key concept of the fictitious rough crack model (FRCM), (I) definition of load path, (II) determination of tensile stresses according to Hillerborg [2], (III) pure shear mode analysis, (IV) and (V) computation of the resulting stresses.

The paper presents a new smeared crack formulation to model shear transfer across cracks and mixed mode fracture of concrete. Aiming at a straight-forward solution, the well-known and easy-to-implement biaxial material model by Darwin and Pecknold [36,37] was used for this purpose, which was combined with the fictitious crack approach of Hillerborg [2] for mode I behaviour of concrete. Additionally, a constitutive model accounting for aggregate interlock of rough crack surfaces (e.g., aggregate interlock model by Walraven [13,14] or Bažant [12]) was applied for mode II behaviour. The main idea of FRCM is to combine these tension-softening (mode I) and shear-transfer laws (mode II) and to superpose the emerging shear and normal stresses of both mechanisms in the crack in order to numerically represent the mixed mode behaviour of concrete. Figure 4 describes the unique procedure and the key concept of the FRCM. The illustrative five-step FRCM procedure for determining the stresses in the crack is as follows.

Step I: The deformation path is given as a function of the two independent parameters of the shear deformation ∆ and the crack opening *w*, illustrated in four different relations of *w*/∆ in Figure 4.
Step II: Tensile stresses in the fracture process zone are initially computed using the fictitious crack model according to Hillerborg [2] (cf. Equation (6)), which is based on the multiaxial tensile strength and the fracture energy in pure mode I condition at crack opening *w*.
Step III: Pure shear mode II is analysed using the relation of *w*/∆ to determine shear and normal stresses in the crack due to aggregate interlock. For this purpose, one can use arbitrary aggregate interlock laws, e.g., approaches by Walraven or Bažant [12,13,14] (cf. Equations (13)–(16)), which allow computing normal and shear stresses in the crack based on a given sliding deformation ∆ and a predefined crack width *w*.
Step IV: In this key step, mode I and II models are combined for mixed mode simulation by superposing the normal stresses of mode I *σ*_Hil_ (tension) with those of mode II *σ*_AI_ (compression). The blue total stress curve is then defined as the superposition of the two curves from the single modes I and II.
Step V: The resulting shear stress curve is established using the shear stresses in the crack computed in step III. Shear stresses can only be induced through mode II deformations, while normal stresses may either be the result of mode I, mode II, or a combination of both.

The model is based on the following main assumptions:

1. The formation of cracks occurs under pure mode I conditions after exceeding the biaxial tensile strength.
2. A fixed crack model is applied.
3. After initial crack opening, Hillerborg’s fictitious crack model is combined with appropriate aggregate interlock models based on the crack surface deformations ∆ and *w*, according to the introduced FRCM procedure.

The proposed model is implemented with the user sub-routine UMAT using Abaqus/Standard and validated by a comparison with current modelling approaches against mode I, mode II, and mixed mode experiments from the literature. The following approaches could also be replaced by other more sophisticated material models without limiting their general applicability.

## 2. Concrete Model

### 2.1. General Equations of the Two-Dimensional Material Model

The implemented two-dimensional concrete model is based on the biaxial material model of Darwin and Pecknold [36,37], which uses equivalent uniaxial principal stress-strain relations for describing the material behaviour. These curves define the principal tangential stiffnesses *E*_1_ and *E*_2_ used for an orthotropic material law (cf. Equation (2)).

(2) $$\begin{array}{l} \left[\begin{array}{c} d\sigma_{1}\\ d\sigma_{2}\\ d\tau_{12} \end{array}\right]=D\cdot \left[\begin{array}{c} d\varepsilon_{1}\\ d\varepsilon_{2}\\ d\gamma_{12} \end{array}\right]\\ =\frac{1}{1-\nu^{2}}\cdot \left[\begin{array}{ccc} \;E_{1} & \nu \cdot \sqrt{E_{1}\cdot E_{2}} & 0\\ \nu \cdot \sqrt{E_{1}\cdot E_{2}} & E_{2} & 0\\ 0 & 0 & \frac{1}{4}\cdot \left(E_{1}+E_{2}-2\cdot \nu \cdot \sqrt{E_{1}\cdot E_{2}}\right) \end{array}\right]\cdot \left[\begin{array}{c} d\varepsilon_{1}\\ d\varepsilon_{2}\\ d\gamma_{12} \end{array}\right] \end{array}$$

*E*_1_ and *E*_2_ are the calculated stiffnesses from the equivalent stress-strain curve in principal direction and *ν* is the Poisson’s ratio, which is set to 0.2 for uncracked and 0 for cracked concrete [38,39] leading to the non-diagonal values becoming zero. The influence of uniaxial compression and biaxial tension-compression states on *ν* due to higher lateral strains [36,37] are neglected. *ε*_1_ is defined as the equivalent maximum principal strain and corresponds to the strain normal to the crack. *ε*_2_ is defined as a minimal principal strain, respectively. *γ*_12_ defines the shear strain, which is null as long as the concrete is uncracked.

The implemented model uses the implicit solving algorithm via the consistent Jacobian in UMAT for solving the tangential stiffness matrix. By using the transformation matrix, the principal stiffness matrix *D* is converted to the axis directions as follows: *D*′ = *T*^T^⋅*D*⋅*T*. The required principal stress angle is calculated by using Mohr’s cycle [40] and the predictor stress, which is introduced in Section 2.2. The transformation matrix is defined in Equation (3) including *θ* being the principle stress angle.

(3) $$T=\left[\begin{array}{c} \;\begin{array}{ccc} \cos{\left(\theta \right)}^{2} & \sin{\left(\theta \right)}^{2} & \sin\left(\theta \right)\cdot \cos\left(\theta \right)\\ \sin{\left(\theta \right)}^{2} & \cos{\left(\theta \right)}^{2} & -\sin\left(\theta \right)\cdot \cos\left(\theta \right)\\ -2\cdot \sin\left(\theta \right)\cdot \cos\left(\theta \right) & 2\cdot \sin\left(\theta \right)\cdot \cos\left(\theta \right) & \cos{\left(\theta \right)}^{2}-\sin{\left(\theta \right)}^{2} \end{array} \end{array}\right]$$

### 2.2. Material Behaviour in Principal Stress Directions

#### 2.2.1. Stress-Strain Relationships

For numerically describing the material behaviour of concrete based on the equivalent stress-strain concept, analytical uniaxial stress-strain relations are needed. For compression, the expression in Equation (4) by Darwin and Pecknold [36,37] is used, which was derived and validated by experimental results of Kupfer, Hilsdorf, and Ruesch [41] as well as Nelissen [42].

(4) $$\sigma =\frac{\varepsilon_{iu}\cdot E_{0}}{1+\left(\frac{E_{0}}{E_{S}}-2\right)\cdot \frac{\varepsilon_{iu}}{\varepsilon_{ic}}+{\left(\frac{\varepsilon_{iu}}{\varepsilon_{ic}}\right)}^{2}}$$

*ε_ic_* is the equivalent strain belonging to the maximum biaxial compressive stress *σ_ic_*, *E*_0_ is the initial tangent stiffness, *E_s_* is the secant stiffness belonging to the maximum biaxial compressive stress *σ_ic_*, and *ε_iu_* is the equivalent strain, which is calculated as input value, according to Equation (5).

(5) $$\varepsilon_{iu}=\int^{\;}\frac{d\sigma_{i}}{E_{i}}=\underset{\begin{array}{c} all\;\\ load\;increments \end{array}}{\sum }\frac{∆\sigma_{i}}{E_{i}}$$

*E_i_* is the tangent stiffness. The equivalent strain concept according to Darwin and Pecknold [36,37] serves to isolate the Poisson effect from the cumulative strains. This allows for determining the true plane biaxial stress-strain state for concrete by using the given equivalent stress-strain relations (e.g., Equation (4)). For determination of the nonlinear stress state, the required *ε_iu_* is calculated at the beginning of the subroutine using a linear elastic predictor stress for ∆*σ_i_*. 

**Figure 5:**
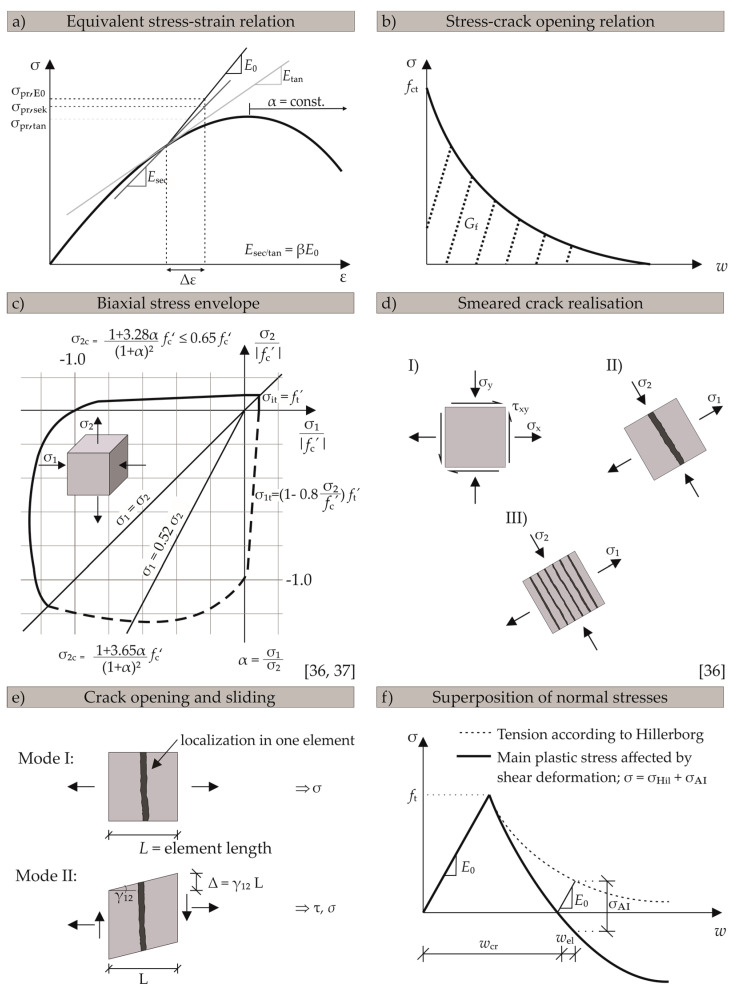
Theoretical background of fictitious rough crack model (FRCM) implementation in UMAT, (**a**) equivalent stress-strain relation, (**b**) typical stress-crack opening relation, (**c**) biaxial strength envelope according to Darwin and Pecknold [36,37], (**d**) smeared crack idealisation according to Darwin and Pecknold [36], (**e**) definition of crack width and shear deformation and (**f**) superposition of normal stresses in the crack.

This stress can be determined by using different approaches for the stiffness matrix, e.g., the initial stiffness *K*_0_, secant stiffness *K*_sec_, or tangential stiffness *K*_tan_ (cf. Figure 5a). The different options were examined. Applying the tangential stiffness was in best accordance with experimental results and resulted in the best results in terms of convergence. Consequently, all results in this paper are based on this option. While material behaviour of uncracked concrete under tensile loads is assumed to be linearly elastic, the definition of cracked tensile behaviour is based on the fictitious crack model of Hillerborg [2]. A stress-displacement (crack width) relation is used to describe the post-cracking behaviour in the fracture zone. To avoid singularity and other numerical problems at the crack tip due to abrupt drop of the stress curve (e.g., [43]), an exponential function (cf. Equation (6)) according to Jirasek [34] is used.

(6) $$\sigma =f_{ct}\cdot e^{-\frac{w\cdot f_{ct}}{G_{f}}}$$

Several approaches for defining fracture energy *G_f_* are available [28,44,45]. In this case, fracture energy is defined as *G_f_* = *G*_f0_⋅(0.1⋅*f*_cm_)^0.7^, according to Model Code 90 [44] while *w* is the crack width, *f_ct_* is the uniaxial tensile strength, and *f*_cm_ is the averaged uniaxial compressive strength. These values are obtained from literature based on experimental investigations.

The basic value *G*_f0_ depends on the maximum aggregate size. Jirasek obtained acceptable results by applying this post-cracking law. If fracture energy properties were available, these documented values were adapted in the numerical simulations presented within this paper. The principal characteristics of the stress-strain curves and stress-crack opening curves are shown in Figure 5a,b, respectively.

#### 2.2.2. Biaxial Mechanical Properties

Concrete behaviour under biaxial stress states is characterised by changing stiffness and strength. In case of biaxial compression, increased stiffness as well as increased strength is observed, which can reach values up to 1.16-times the uniaxial strength. The influence of biaxial stress states is taken into account using Kupfer’s [46] modified [36,37] analytical strength envelope shown in Figure 5c.

The biaxial strength values are, thus, calculated depending on the stress state, which is described by *α* = *σ*_1_/*σ*_2_. This is the ratio of the maximum to the minimum stress. The variation of ductility of concrete due to biaxial stress sates is considered by two criteria for a biaxial compressive strength being either larger (case 1) or smaller (case 2) than the uniaxial compressive strength [36,37].

(7) $$\sigma_{ic}<f_{c}^{\prime }\;\varepsilon_{ic}=\varepsilon_{cu}\cdot \left[-1.6\cdot {\left(\frac{\sigma_{ic}}{f_{c}^{\prime }}\right)}^{3}+2.25\cdot {\left(\frac{\sigma_{ic}}{f_{c}^{\prime }}\right)}^{2}+0.35\cdot \left(\frac{\sigma_{ic}}{f_{c}^{\prime }}\right)\right]$$

(8) $$\sigma_{ic}\;\geq \;f_{c}^{\prime }\;\varepsilon_{ic}=\varepsilon_{cu}\cdot \left[\frac{\sigma_{ic}}{f_{c}^{\prime }}\cdot R-\left(R-1\right)\right]$$

where

(9) $$R=\frac{\frac{\varepsilon_{ic}\left(\alpha =1\right)}{\varepsilon_{cu}}-1}{\frac{\sigma_{ic\left(\alpha =1\right)}}{f_{c}^{\prime }}-1}\approx 3.0$$

*ε_cu_* is the uniaxial fracture strain and *f_c_*′ is the uniaxial compressive strength. The value of *R* ≈ 3 is based on the experimental investigations of Kupfer [41], Liu [47], and Nelissen [42] and was used for the concrete model.

### 2.3. Modelling of Cracked Concrete Behaviour

The cracked concrete behaviour is realised by adopting Equation (2) to an orthotropic constitutive law based on smeared fixed crack model approaches resulting in Equation (10).

(10) $$D=\left[\begin{array}{c} \;\begin{array}{ccc} E_{1} & 0 & 0\\ 0 & E_{2} & 0\\ 0 & 0 & \frac{E_{2}}{4} \end{array} \end{array}\right]\cdot \left[\begin{array}{c} d\varepsilon_{1}\\ d\varepsilon_{2}\\ d\gamma_{12} \end{array}\right]$$

*E*_1_ and *E*_2_ are the tangential stiffnesses in principal axis directions with Poisson’s ratio being zero. This tangential matrix is used for incremental calculation of the predictor step while the Jacobian matrix needed for the implicit solving algorithm is numerically determined. The nonlinear stresses are computed, according to the given stress-strain relations in Section 2.2.

If the biaxial tensile strength is exceeded, the single crack is idealised as smeared along one element, according to Reference [36], as shown in Figure 5d. The FRCM uses a fixed crack approach with two possible crack directions. To realise an accurate representation of concrete behaviour under mixed mode conditions, aggregate interlock models of Walraven [13,14] and Bažant [12] are used to describe the shear stresses and the resulting compressive normal stresses. Both models are in compliance with the theory regarding the interaction between normal and shear deformations. The smeared definition of shear deformation can be defined by referring to a discrete description of a crack (cf. Figure 5e). Due to the description of post cracking behaviour in fixed crack approaches, shear displacements occur. Shear displacement is defined as a product of the shear angle *γ*_12_ with the element length *L*. Due to negative normal stresses caused by aggregate interlock, assessing the crack width is more complex compared to pure mode I behaviour [2]. In order to make use of post cracking behaviour, the compressive normal stress σ_AI_ resulting from aggregate interlock has to be subtracted from the total normal stress (cf. Figure 5f).

This key concept of FRCM (cf. Figure 4) is realised via superposition of the resulting tensile stresses of mode I, according to Hillerborg, and mode II, according to aggregate interlock models (cf. Equation (11)).

(11) $$\sigma_{N}{=\sigma }_{\mathrm{Hil}}{+\sigma }_{\mathrm{AI}}$$

(12) $$∆=\gamma_{12}\;\cdot \;L\;\mathrm{with}\;L=\mathrm{Element}\;\mathrm{length}$$

Crack formation control by pure mode I through the stress-crack opening displacement curve [2,4,5,39] does not cause mesh dependencies when defining shear deformation of the crack faces as the element length (cf. Equation (12)). Different approaches can be used for defining the transmittable stresses σ_AI_ due to aggregate interlock. Walraven [13,14], for example, used a probability distribution of the aggregates in the concrete as well as a Fuller curve. His complex approach, therefore, considers the maximum aggregate size, the matrix strength, and, consequently, the crucial influence of the concrete composition on the mechanism of aggregate interlock. In this paper, Walraven’s simplified formulae (cf. Equations (13) and (14)) based on his experimental results are considered.

(13) $$\tau =-\frac{f_{\;\mathrm{cc}}^{\;\prime }}{30}+\left[1.8\;\cdot {\;w}^{-0.8}+(0.234\;\cdot {\;w}^{-0.707}-\;0.20)\cdot {\;f}_{\;\mathrm{cc}}^{\;\prime }\right]\cdot ∆\;>\;0$$

(14) $$\sigma =-\frac{f_{\;\mathrm{cc}}^{\;\prime }}{20}+\left[1.35\;\cdot {\;w}^{-0.63}+(0.191\;\cdot {\;w}^{-0.552}-\;0.15)\;\cdot {\;f}_{\;\mathrm{cc}}^{\;\prime }\right]\cdot \;∆\;>\;0$$

In this case, *f*^′^_cc_ is the cube compressive strength, *w* is the crack width, and ∆ is the shear deformation. These equations are defined for maximum aggregate sizes between 16 and 32 mm.

Bažant’s [4] model was derived from Paulay’s [10] experimental test data with maximum aggregate size of 19 mm, according to Equations (15) and (16).

(15) $$\sigma_{\;\mathrm{nt}}^{\;c}{=\tau =\tau }_{u}\;\cdot \;r\;\cdot \;\frac{a_{3}{+a}_{4}\;\cdot \;{\left|r\;\right|}^{3}}{{1+a}_{4}\;\cdot {\;r\;}^{4}}$$

(16) $$\sigma_{\;\mathrm{nn}}^{\;c}=\sigma =-\frac{a_{1}}{\delta_{n}}\;\cdot \;{\left(a_{2}\;\cdot \;\left|\sigma_{\;\mathrm{nt}}^{\;c}\right|\right)}^{p}$$

Equations (15) and (16) (Notations adopted from Bažant) mainly depend on the ratio *r* between shear and normal deformation as well as the maximum shear stress *τ*_u_ = 0.245⋅*f*_c_^′^ (*a*_1_ to *a*_4_ are constants). Verified aggregate interlock models for concrete or mortar compositions with smaller maximum aggregate sizes are lacking.

## 3. Validation of the Proposed Model

### 3.1. General

Solving the differential equations is based on the discretisation of a continuum into finite elements with suitable selection of approaches. The available elements in Abaqus differ in terms of the element type and the order of Ansatz-function. The plane stress element CPS4R with reduced integration is used in Abaqus for modelling different experimental investigations for validation.

### 3.2. Comparison with Analytical Solutions for One Single Element

The first step to check the implemented material model was to validate the implemented constitutive equations by applying different load combinations on one single 5 mm × 5 mm finite element. Material parameters of *E* = 30,000 N/mm^2^, *f*_c,cyl_ = 25 N/mm^2^, *f_ct_* = 3 N/mm^2^, *G_f_* = 0.06 N/mm, and a maximum uniaxial concrete compressive strain of *ε_cu_* = 2.2‰ are used. Displacement loads are applied for verification of descending branch for the stress ratio to be transformed into a strain ratio *ε*_1_/*ε*_2_ = (*α*−*ν*)/(1−*α*⋅*ν*) by using the stiffness matrix in Equation (2) and assuming *E*_1_ = *E*_2_. The main findings are presented in the following for different load combinations.

#### 3.2.1. Biaxial Material Behaviour in Principal Stress Directions

**Figure 6:**
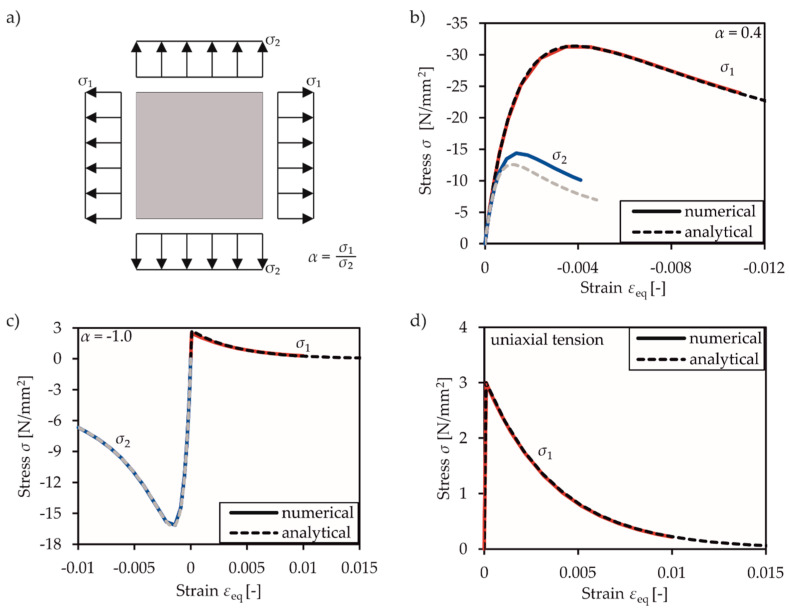
Illustration of (**a**) principal stress state, and of numerical verification results compared to analytic solution for (**b**) compression-compression, (**c**) tension-compression, and (**d**) uniaxial tension.

For validation of the material behaviour under principal stress conditions, different principal stress ratios *α* are assessed (cf. Figure 6a). The result for *α* = 0.4 is shown as a representative example for the biaxial compression state in Figure 6b. The illustrated numerical result is in perfect accordance with the analytical equations for the main compressive stress. The stresses of the minor direction show an overestimation of 15%. This can be explained by inaccuracies in determining the tangential stiffness matrix *K*_tan_, which is used for predictor stress and affects the main stress ratio.

In case of compression-tension and uniaxial tension (cf. Figure 6c,d), there was no difference between the numerical and analytical solutions. This can be explained by the early stage of reaching the maximum tensile strength *f_ct_*, as long as the material behaviour remains linearly elastic.

#### 3.2.2. Shear and Mixed Mode Behaviour

**Figure 7:**
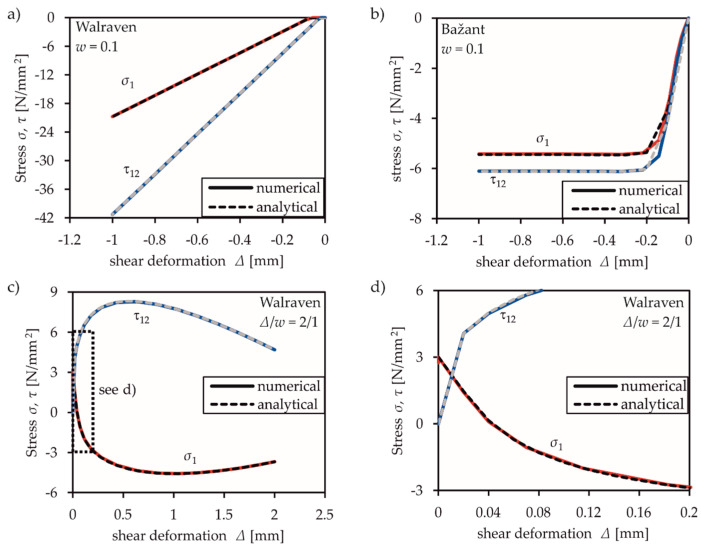
Illustration of numerical verification results compared to a theoretically analytic solution for (**a**) and (**b**) shear with constant crack width and (**c**) and (**d**) constant tension-shear condition., FRCM with Walraven’s or Bažant’s aggregate interlock model.

To validate the implemented shear and mixed mode behaviour based on the combination of mode I and II, two different load cases were examined. Before applying pure shear mode, the element was first cracked in mode I and the crack was opened until reaching a crack width of 0.1 mm. Then, pure shear loading was applied while maintaining a constant crack width. The results based on Walraven’s (cf. Figure 7a) and Bažant’s (cf. Figure 7b) aggregate interlock model both agree with the analytical solutions. The influence of the linear regression formulae by Walraven [13,14] is clearly visibly in Figure 7a. These formulae have no upper limit of transferable shear stress. The application of Bažant’s rough crack model [4] in FRCM in Figure 7b considers an upper limit and, therefore, leads to a more representative behaviour.

Nonetheless, in real load conditions, this upper limit value is likely not decisive, as shown in Figure 7c,d. The element was first loaded until the tensile strength was reached. Then, a constant relation between normal and shear deformation was applied to assume the expected mixed mode conditions. Mixed mode loading paths with shear and normal displacements in proportion ∆/*w* = 2/1 cause a parabolic curve with a descending branch in dependence of the interaction between ∆ and *w*. With increasing crack width *w*, the transmittable stresses decrease as expected. The numerical solution is in perfect agreement with the analytical solution, which can be determined in the same way as the numerical one, according to Figure 4.

The FRCM allows different possibilities to determine aggregate interlock behaviour of concrete and, hence, enables it to react to different concrete compositions when theoretical models are available.

### 3.3. Validation with Benchmark Tests

**Figure 8:**
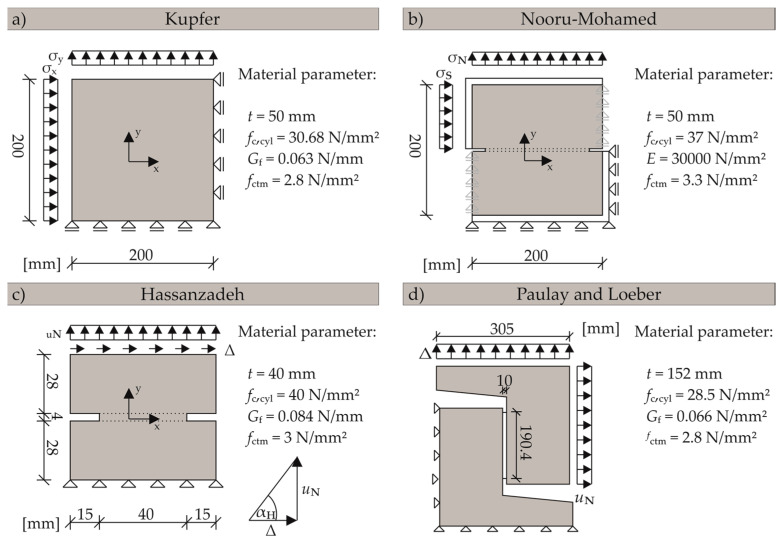
Illustration of the numerical boundary conditions and the material parameters, (**a**) Kupfer et al. [41], (**b**) Nooru-Mohamed [20], (**c**) Hassanzadeh [19] and (**d**) Paulay and Loeber [10].

Additionally, different experimental investigations were recalculated to validate the implemented FRCM. An overview of these experiments and the used material parameters are shown in Figure 8. All validations were conducted with a discretisation of the entire experimental specimens using a mesh size between 4 and 10 mm based on the specimen sizes. The load was applied with a maximum increment size of 0.1, while the lower limit was set to 10^−30^.

#### 3.3.1. Behaviour under Normal Stresses: Biaxial Stress State Experiments of Kupfer

The experiments of Kupfer [41] are used for verification of biaxial material behaviour of the proposed model. The measurements and the material parameters of the experiments are shown in Figure 8a.

**Figure 9:**
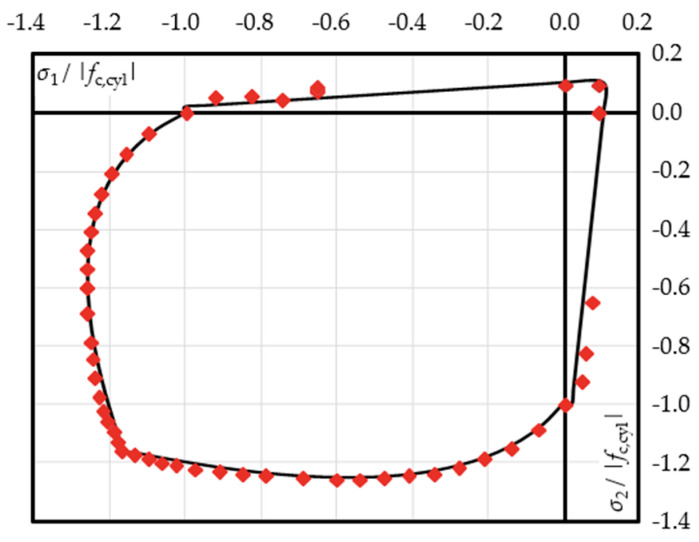
Numerical results compared to the strength envelope, according to Kupfer et al. [41].

The simulation results are compared against Kupfer’s biaxial strength envelope in Figure 9.

The maximum stresses obtained from the experiment are slightly overestimated for the tension-compression case. The overestimation increases with a growing *α*-ratio (cf. Section 2.2.1).

This is due to the minimum compressive strength of 0.65⋅*f*_c,cyl_ according to Darwin and Pecknold’s [36] modified strength envelope (cf. Figure 5c), which acts like a lower limit of 65% of uniaxial compressive strength for increasing tensile stress while the tensile strength increases for decreasing compressive stresses. Besides the consequently increased numerical stresses at *σ*_1_/*f*_c,cyl_ = −0.65, perfect correspondence to the modified strength envelope can be recognised.

#### 3.3.2. Mixed Mode Behaviour and Interaction of Shear and Normal Deformations

This section comprises modelling of three well known mixed modes and mode II experiments with FRCM. The mixed mode experiments are notched panel tests by Nooru-Mohamed [20] and Hassanzadeh [19]. The verification of the FRCM is completed by numerically representing the experiments of Paulay and Loeber [10] with a constant crack width.

(1) Mixed mode experiments by Nooru-Mohamed

Nooru-Mohamed [20] investigated concrete behaviour under mixed mode loads in 1992 (cf. Figure 2a). Concrete compositions with a maximum aggregate size of 2 mm were predominantly used in these experiments. Various combinations of tensile and shear loads were used to experimentally determine different crack patterns. The loads were applied successively (first shear load followed by normal deformation). The boundary conditions for the numerical model and the general material parameters are shown in Figure 8b.

**Figure 10:**
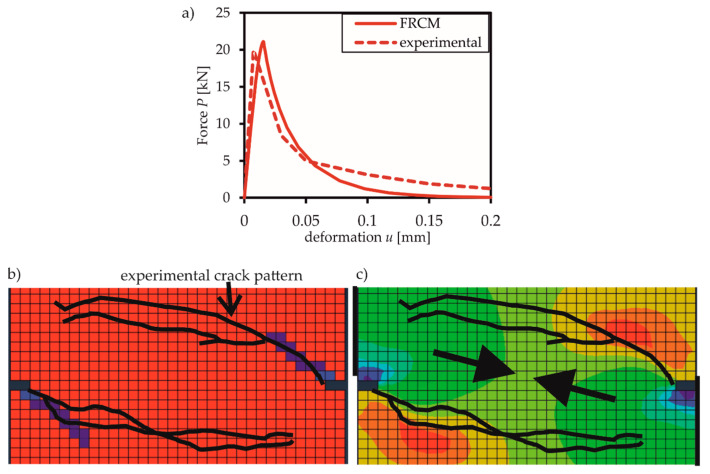
Illustration of (**a**) numerical force-deformation curve for load path 0, (**b**) numerical crack pattern illustrated with the damage parameter *β* for the elasticity modulus, and (**c**) numerical main stress condition. All were compared to the experimental results of Nooru-Mohamed [20] by using Walraven’s [13,14] aggregate interlock model.

The results of load path 0 (pure tension) in Figure 10a show that the experimental and simulated stress-deformation curves are in good agreement.

The numerical crack pattern as well as the principal stresses for load path *4b* (first loading with a constant shear force of 10 kN followed by loading of tensile deformation) obtained with Walraven’s [13,14] aggregate interlock model are shown in Figure 10b,c as compared to the experimental results. The crack is beginning in the notched part and is then developing at an angle of nearly 45° to both sides of the panel. Between the two cracks, a compressive strut develops for transmitting the shear forces (cf. Figure 10c). The numerical crack pattern is less developed than the experimental one. The reason lies in the termination of the simulation prior to the final experimental state due to numerical convergence problems. However, the phenomenology of cracking can be reproduced satisfactorily. One possible reason for the premature termination of the simulation could be the lacking of adequate constitutive relations for aggregate interlock. The crack-friction laws by Walraven and Bažant, which have been used for simulation, are not applicable to the small aggregate size of 2 mm of Nooru-Mohamed’s experiments. These inadequate crack-friction laws may have led to a delayed activation of shear stresses in the crack. In consequence, there was an extended phase without significant shear stress transfer, which could have resulted in a premature abortion of the simulation. In contrast to the aggregate interlock behaviour, where adequate constitutive relations for a 2 mm maximum aggregate size are missing, *G_f_* was varied to account for the small aggregate sizes. According to Reference [44], a suitable value for a maximum aggregate diameter of 2 mm is 0.06 N/mm.

(2) Mixed mode experiments by Hassanzadeh

In this section, the simulations of the mixed mode experiments by Hassanzadeh [19] to investigate concrete behaviour under combined loading of normal and shear deformations are described. The main focus of this work is the representation of complex crack propagation mechanisms under mixed mode conditions. The numerical conditions as well as the material parameters and the boundary conditions are shown in Figure 8c.

**Figure 11:**
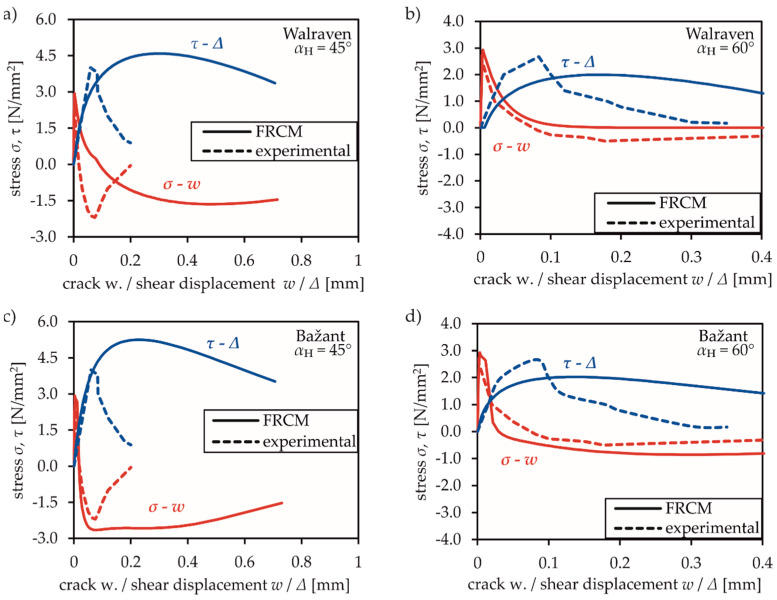
Comparison of numerical stress-displacement curves (FRCM) with experimental results, FRCM with Walraven for (**a**) *α*_H_ = 45° and (**b**) *α*_H_ = 60°, FRCM with Bažant for (**c**) *α*_H_ = 45° and (**d**) *α*_H_ = 60°.

In contrast to the tests by Nooru-Mohamed, the specimens were loaded until tensile strength was reached in the first step. In the second step, various ratios of simultaneously acting shear and normal deformation were applied, which led to crack development under combined actions. The test setup produces one single crack, which allows analysing the stress-strain relations in one specific material point. Validation in this study is performed via tests with a maximum aggregate size of 8 mm, which were loaded by a linear deformation ratio whose angle *α*_H_ between normal and shear deformation varied between 30° and 75°. Figure 11 shows the experimental results in comparison to the numerical FRCM results. *σ* and *τ* were evaluated by determining the reaction forces in the numerical model and relating them to the sheared filament.

Figure 11 illustrates the normal stress over the crack opening and the shear stress related to the shear deformation for FRCM using the empirical approach by Walraven [13,14] (cf. Figure 11a,b) and the rough crack model by Bažant [12] (cf. Figure 11c,d). In principle, reasonable agreement can be observed in all diagrams. The deviations are due to inappropriate constitutive relations of crack friction for concrete mixes with small grain sizes. On the one hand, FRCM with Walraven’s material model considers larger maximum aggregate diameters leading to higher transferred stresses in the crack even at large crack widths. This might be the reason for the overestimation of shear and normal stresses in Figure 11a with a significant shear component (*α*_H_ = 45°). On the other hand, a larger slip occurs due to the larger aggregates and, consequently, larger shear deformations are required to obtain shear stresses. Thus, in tests with dominant mode I deformation of crack surfaces (e.g., Figure 11b, *α*_H_ = 60°) and small shear deformation, this effect leads to an underestimation of the transferable stresses.

The results of FRCM with Bažant’s model show an adequate estimation of the maximum transferable stresses for both load angles (cf. Figure 11c,d). Both models show a ductile post-cracking behaviour but do not consequently capture the stress drop of experimental investigations in the post-failure range.

Generally, one can see the ability to adapt the mixed mode behaviour of concrete with the FRCM approach. This lack only exists in the constitutive modelling of aggregate interlock for different concrete compositions. There is still a lot of experimental work required to characterise adequate aggregate interlock laws for concrete mixtures with small maximum aggregate sizes. Therefore, a novel experimental procedure based on torsion testing has been proposed in Reference [1]. It was used to conduct initial tests to later determine aggregate interlock laws for fine grain concretes based on more extensive test series.

#### 3.3.3. Aggregate Interlock Experiments of Paulay

To confirm that the FRCM in combination with aggregate interlock laws of Walraven [13,14] and Bažant [12] is able to adequately capture the behaviour of tests with medium and large aggregate diameters, a numerical simulation of the experimental investigations of Paulay and Loeber [10] with an aggregate size of 19 mm were performed. Forty-four specimens were tested in 1974 to investigate the crack friction mechanism. The crack opening was kept constant in the tests while loading was applied as shear deformation. The concrete composition corresponds to that of Walraven [13,14]. In addition, Bažant’s [12] aggregate interlock law was derived from these test results. The experimental set up is shown in Figure 8d.

**Figure 12:**
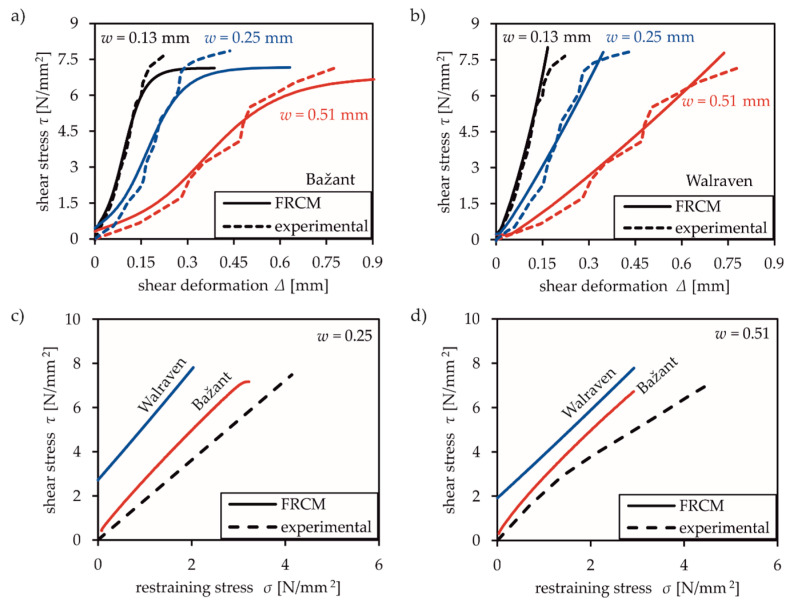
Comparison of numerical results with FRCM (aggregate interlock models by Walraven and Bažant) and experimental results, (**a**) shear stress-deformation for Bažant, (**b**) shear stress-deformation for Walraven, (**c**,**d**) relation of shear stress and restraining stress.

Figure 12 illustrates the experimental results by Paulay for different crack openings. Figure 12a,b show the relation between transferable shear stress and shear deformation while Figure 12c,d show the resulting normal or restraining compressive stresses in relation to the shear stresses. The experimental results represent the averaged values, where the shear deformations were experimentally determined above and below the crack. Using Bažant’s aggregate interlock law (cf. Figure 12a), FRCM predicts the transferred shear stresses as well as the resulting normal compressive stresses for all crack openings with reasonable agreement. This also applies to the numerical results where Walraven’s aggregate interlock law was applied (cf. Figure 12b). For the effect of compressive normal stresses, illustrated in Figure 12c,d, the usage of FRCM with Bažant’s aggregate interlock model gives the best prediction of experimental results, while Walraven’s model requires higher shear deformations to activate compressive stresses. Reviewing the numerical predictions of Nooru-Mohamed’s [20] and Hassanzadeh’s [19] test data shows that inappropriate laws for small aggregate sizes (smaller than 8 mm) seem to be the reason for occurring deviations.

## 4. Conclusions

The paper presents a two-dimensional numerical material model named fictitious rough crack model (FRCM) for prediction of mode II and mixed mode behaviour of concrete. The FRCM, implemented in Abaqus by user subroutine UMAT, considers a new smeared crack formulation based on Darwin and Pecknolds’ biaxial material model. It is extended by the fictitious crack model by Hillerborg and constitutive models for aggregate interlock by Walraven’s two phase model or Bažant’s rough crack model. The model, therefore, includes the following characteristics of concrete behaviour.

1. Biaxial failure criteria,
2. Variation of tensile and compressive strengths as well as stiffnesses under biaxial loading conditions,
3. Prediction of the softening branch of uniaxial stress-strain relations of tension via fracture energy criterion and compression via analytical stress-strain relations of Darwin and Pecknold,
4. Representation of aggregate interlock effects in shear and normal direction after formation of mode I cracks.

A review of frequently used numerical models is presented and validated for aggregate interlock behaviour of concrete by applying these models to the benchmark test of Paulay and Loeber with a constant crack width. After comparison of numerical results by FRCM and commercial models with test data, the following conclusions can be drawn.

1. The commercial models (Abaqus CDP, ATENA SBETA, and ATENA Microplane4) show a significant lack of prediction of aggregate interlock effects due to over-simplification of the underlying mechanisms.
2. The use of shear retention factors for shear modulus-crack width relation leads to unsatisfactory results for mixed mode cracking and does not solve the problem of complex interaction of crack width and shear deformation in case of mixed mode behaviour of concrete.
3. In contrast, the FRCM approach seems to adequately capture the fundamental principles of mode II and mixed mode behaviour and shows reasonable agreement with experimental results.
4. The validation of experimental results of Nooru-Mohamed and Hassanzadeh with a maximum aggregate size of 8 mm is clouded by the fact that adequate constitutive relations for concrete mixtures with fine aggregates and mortars are not available. Clearly, concrete composition, distribution of aggregates, and a maximum aggregate size significantly influence the shear response and lead to certain deviations between experimental and numerical results. On the one hand, a large aggregate diameter leads to higher transferable stresses while, on the other hand, it requires higher values of shear deformation (slip) to activate a specific stress level in the crack.
5. The crack friction or mixed mode behaviour is decisively influenced by the behaviour of the crack surfaces. Opposing simplified approaches, the interaction of shear and normal deformation is of particular significance for assessing the transferred stresses in the fracture process zone.

The intention of the presented study was to improve the understanding of aggregate interlock effects in mixed mode cracking and to clarify the unsatisfying consideration in common models of commercial software. After comparing the numerical results to experimental data, there is no doubt about the improved prediction of aggregate interlock effects with FRCM. With the exception of the microplane model, the investigated models (CDP, ATENA, SBETA, or Microplane M4) are not able to realistically represent the activation of shear and compressive normal stresses. The frequently used CDP-model completely neglects aggregate interlock effects. In contrast, the microplane M4 model delivers relevant shear and compressive normal stresses in the crack. However, microplane models do not address the real underlying mechanisms of aggregate interlock since shear and compressive normal stresses are the outcome of superposing mode I behaviour of several microplanes. Due to the lack of realistic consideration, these models also lead to considerable deviations.

In the past, less attention was paid to the representation of shear and mixed-mode behaviour of concrete while the usage of finite elements started to gain more importance. Regarding the increased research on shear in reinforced concrete structures [48] and their numerical prediction, the lack of consideration of aggregate interlock is of crucial importance since it is recognised to have a decisive influence on shear capacity. The results shown in this paper corroborate that common commercial models are not appropriate to solve the problem for predominantly shear loaded concrete components due to the missing consideration of aggregate interlock mechanism.

The FRCM is a first and humble approach to deepen the understanding of aggregate interlock effects. The modern technical possibilities within FEM and the achieved results with FRCM show the potential of a refined numerical analysis of the mixed mode behaviour of concrete. This paper is intended to be a motivation for future studies in the theoretical principles of aggregate interlock and mixed mode behaviour and their refined consideration in analytical and numerical models. 

## Figures and Tables

**Table 1:** Rating of material models against different criteria.

Model		Concrete Damaged Plasticity	ATENA Constitutive Model SBETA (CCSbetaMaterial)	ATENA Microplane Material Model (CCMicroplane4)
Nonlinear tension behaviour		+	+	+
Nonlinear compression behaviour		+	+	+
Biaxial concrete behaviour		+	+	+
Aggregate interlock	Normal	−	−	o
	Shear	−	o	o
Crack model		R	F or R	/

+ well considered; o simplified consideration; − unconsidered; F = fixed; R = rotated; / not rateable.

**Table 2:** Parameters for the numerical simulations with concrete damaged plasticity (CDP).

Concrete Damaged Plasticity [33]			
*E*	30,000 N/mm^2^	Tension softening Jirásek [34]	$\sigma =f_{ct}\cdot e^{-\frac{w}{w_{1}}}$
*ν*	0.2	Compression Sargin [35]	$\sigma_{c}{=f}_{\mathrm{cm}}\;\cdot \;\frac{k\;\cdot \;\eta \;-\;(D\;-\;1)\;\cdot {\;\eta }^{2}}{1+\left(k\;-\;2\right)\;\cdot \;\eta +D\;\cdot {\;\eta }^{2}}$
Dilation angle *ψ*	35°	*D*	0.4
Eccentricity *ε*	0.1	η	$\varepsilon_{c}/\;\varepsilon_{c1}$
*σ*_b0_/*σ*_c0_	1.16	k	$E_{c0}\;\cdot {\;\varepsilon }_{c1}/\;f_{\mathrm{cm}}$
*K*_c_	0.68	*ε*_c1_	strain at maximum stress

**Table 3:** Parameters for the numerical simulations with ATENA SBETA.

ATENA SBETA, Fixed Crack Model [32]			
*E*	30,000 N/mm^2^	Tension softening	$w_{c}=5.14\;\cdot \;\frac{G_{f}}{f_{t}}$
*ν*	0.2	Compression softening	*w*_d_ = −0.5 mm
*ε_cu_*	−2.2‰	Shear retention factor	Variable (logarithmic)
*c*	0.8	Tension-compression interaction	linear

**Table 4:** Parameters for the numerical simulations with ATENA Microplane M4.

ATENA Microplane M4 [7]			
*E*	30,000 N/mm^2^	K_3_	15
*ν*	0.18	K_4_	150
K_1_	1.1168⋅10^−4^	K_5_	0.0001
K_2_	500	Number of microplanes	37
		Crack band size	0.03 m
